# Characterization of a novel murine model of *Toxoplasma gondii* infection using oocysts of a recently obtained Type III isolate

**DOI:** 10.1186/s13567-025-01507-x

**Published:** 2025-04-02

**Authors:** Andrea Largo-de la Torre, Ignacio Ferre, Roberto Sánchez-Sánchez, Javier Regidor-Cerrillo, Luis Miguel Ortega-Mora

**Affiliations:** 1 SALUVET-Innova, Faculty of Veterinary Sciences, Complutense University of Madrid, Ciudad Universitaria s/n, 28040 Madrid, Spain; 2 SALUVET, Animal Health Department, Faculty of Veterinary Sciences, Complutense University of Madrid, Ciudad Universitaria s/n, 28040 Madrid, Spain

**Keywords:** *Toxoplasma gondii*, mouse model, oocyst, Type II and Type III, field isolate

## Abstract

Type II reference isolates of *Toxoplasma gondii* are widely used in animal toxoplasmosis models, but studies with Type III isolates remain scarce. In addition, these methods often rely on laboratory-adapted parasite stages that may not reflect natural infection. This study presents a new murine model based on an oral infection with oocysts from a recently obtained Type III isolate, TgShSp24, which exhibited remarkable morbidity and a distinct tissue distribution during chronic infection, differing from the recently obtained Type II isolate TgShSp1. This novel model aims to better mimic natural infection and provides a valuable tool for testing drugs and vaccines.

## Introduction, methods and results

### Importance of well-characterized murine models for studying *T. gondii* infection

*Toxoplasma gondii* is a protozoan parasite responsible for toxoplasmosis, a zoonotic disease that affects approximately one-third of the global population. Infection occurs in all warm-blooded animals, including humans, and is transmitted primarily through the ingestion of oocysts from contaminated sources, through undercooked meat containing tissue cysts, or via vertical transmission [1]. The parasite’s life cycle involves sexual reproduction in felids and asexual reproduction in intermediate hosts, leading to the formation of cysts in neural and muscular tissues. While infections are often asymptomatic in immunocompetent individuals, toxoplasmosis can result in severe conditions such as toxoplasmic encephalitis in immunocompromised hosts. Congenital cases of toxoplasmosis may result in miscarriage or long-term health complications [2].

Murine models have been indispensable in advancing our knowledge of toxoplasmosis, particularly in elucidating the immunological and molecular mechanisms involved in the transition from acute to chronic infection, tissue cyst formation, and host immune response to *T. gondii*, as well as in establishing models for evaluating the efficacy of potential drugs and vaccines [3–7]. However, established models often rely on laboratory-adapted *T. gondii* isolates that have undergone an undetermined number of in vitro passages, potentially altering their virulence and failing to accurately reflect the behavior of field isolates [8]. Moreover, *T. gondii* infection has been extensively studied using tachyzoites introduced intraperitoneally or subcutaneously into mice because of the ease of laboratory maintenance, but these routes do not reflect natural infection pathways [9]. The most widely used laboratory-adapted strains are Type II isolates, such as ME49 and PRU, and they have been used to study chronic infection in mice and to test vaccines and drugs. In contrast, studies involving Type III isolates using oocysts remain very limited [5, 10].

In this study, we report a Swiss CD1 murine model infected with oocysts from the recently obtained TgShSp24 isolate. The *T. gondii* Type III (genotype #2) isolate TgShSp24 was obtained from the myocardium of chronically infected adult sheep (4–5 years old) of Manchega × Lacaune breed, located in the province of Ciudad Real (central Spain) [11]. The model was also compared with infection using oocysts from TgShSp1, a recently obtained Type II (genotype #3) isolate from a *T. gondii* ovine abortion outbreak in a Spanish sheep flock (Assaf breed) in the province of Palencia (northwest Spain) [11].

### Experimental design and analyses for the development of a toxoplasmosis mouse model

This study was carried out with an in vitro, controlled-passage field isolate of *T. gondii*, TgShSp24 (Type III, ToxoDB genotype #2), which was compared with the well-characterized TgShSp1 (Type II, ToxoDB genotype #3), controlled-passage. The isolates were used at low cell culture passages, ranging from 8 to 15 passages.

**Figure 1:**
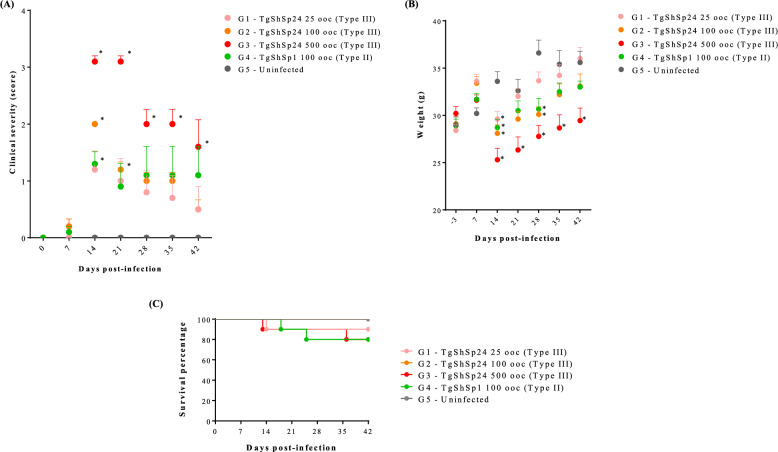
**Morbidity and mortality in mice infected with oocysts from the TgShSp1 and TgShSp24 isolates.** Severity of clinical signs (**A**) according to assigned score: asymptomatic (= 0), ruffled coat (= 1), rounded back (= 2), weight loss/body condition (= 3), and presence of neurological signs/sudden death (= 4), body weight graph (**B**), and Kaplan‒Meier survival curve (C). The curve illustrates the probability of survival over time for each group, providing a stepwise representation of survival rates. Each point in (**A**) and (**B**) represents the mean value, and the bars indicate the standard error. Mortality rates were compared using the Mantel‒Cox log-rank test, body weights by one-way ANOVA followed by Tukey’s multiple comparisons test, and variation in morbidity scores via two-way ANOVA. * represents significant differences in the infected group compared with the control uninfected group, *p* < 0.05 (**C**).

All animal procedures were approved by the Animal Welfare Committee of the Community of Madrid, Spain (PROEX 290.4/20 and PROEX 062/19), following Spanish and EU regulations (Law 3/2007, R.D. 53/2013, and Council Directive 2010/63/EU). Forty-five 6-week-old female Swiss/CD1 mice (Janvier Labs, Le Genest-Saint-Isle, France) were used for the experimental infection and housed in a controlled environment under a 12-h light/dark cycle, with rodent feed and water ad libitum. Following a 7-day adaptation period, the mice were randomly allocated into five groups: four groups of 10 animals each (G1, G2, G3, and G4) and one group of five animals (G5). Mice were orally inoculated with three different doses of TgShSp24 (Type III) to study infection: 25 (G1), 100 (G2) and 500 (G3) oocysts. To compare the effects of strain type at the same dose and as an internal control to check reproducibility, Group G4 was dosed with 100 oocysts of TgShSp1 (Type II), as previously described [12]. Oocysts were obtained through the oral infection of cats [12]. Briefly, Swiss /CD1 mice were intraperitoneally inoculated with cell culture-derived tachyzoites (500 tachyzoites TgShSp24 and 1000 tachyzoites TgShSp1, in accordance with their virulence degree in mice), which had been maintained at low cell culture passage (n < 15). Two months post-inoculation, the mice were euthanized, and their brains were collected to feed the kittens. The oocysts were maintained at 4 °C until inoculum preparation within the same year of production. For inoculum preparation, sporulated oocysts were quantified using a single-use Neubauer chamber (DHC-N01 Neubauer Improved CYTO, Gentaur, UK) and subsequently diluted in PBS to the required concentrations for each group. The control group (G5) was inoculated with PBS. The mice were examined twice daily throughout the experiment for clinical signs compatible with toxoplasmosis, conveniently scored (see figure caption in Figure 1), and weighed weekly. Animals that displayed severe loss of body condition/weight (≥ 20%), respiratory distress, or neurological signs were humanely euthanized. All remaining asymptomatic mice were euthanized 42 days post-infection [9].

Infection dynamics were evaluated by *T. gondii* DNA detection and quantification in target organs: the brain, lung, heart, and tongue, as wells as the masseter, quadriceps, and *longissimus dorsi* muscles, as previously described [8]. Additionally, the number of cysts in the brain was determined by fluorescence-conjugated *Dolichos biflorus* lectin (DBL) (Vector Laboratories) staining and counting via microscopy [8]. The IgG immune response was evaluated in serum samples collected via intracardiac puncture by ELISA as previously described [8, 13]. The secondary antibodies used were specific monoclonal antibodies against mouse IgG (1:10 000; A9044, Sigma Aldrich, Madrid, Spain), IgG1 (1:5000; 1080-05, SouthernBiotech, Birmingham, USA), and IgG2 (1:1000; 1070-05, Southern Biotech, Birmingham, USA) conjugated with peroxidase enzyme. All the statistical analyses and graphical illustrations were performed using GraphPad Prism 6 v.6.01 software (San Diego, CA, USA).

### The Type III TgShSp24 isolate results in chronic infection in mice

In all infected groups, during the first week of follow-up post infection, only mild clinical signs, such as ruffled coats, were observed in a few mice, regardless of the oocyst dose (Figure 1A). The severity of the clinical signs increased during the second week, when the majority of the infected mice exhibited ruffled coats, potentially related to fever, and rounded backs, particularly in the G2 and G3 groups, which received the highest infection doses (100 and 500 oocysts, respectively). These behaviors were accompanied by significant weight loss (*p* < 0.05, one-way ANOVA followed by Tukey’s multiple comparisons test) (Figures 1A and B). Despite twice daily monitoring and thorough clinical follow-up of the animals, two sudden deaths occurred on days 13 and 14 post-infection in Groups G1 (25 oocysts) and G3 (500 oocysts), respectively, both of which involved the mice with the lowest body weights within their respective groups. By the third week, most clinical signs had resolved in the majority of mice, with only ruffled coats persisting until the end of the experiment (Figure 1A). Mice in the group infected with 500 oocysts (G3—Type III) were the most affected by the end of the trial (*p* < 0.05, two-way ANOVA) (Figure 1A). One mouse from Group G3 was euthanized on day 38 post-infection because of the onset of neurological signs. However, no significant differences in survival time were observed between the groups (Figure 1C) (*p* > 0.05, Mantel‒Cox log-rank test).

**Figure 2:**
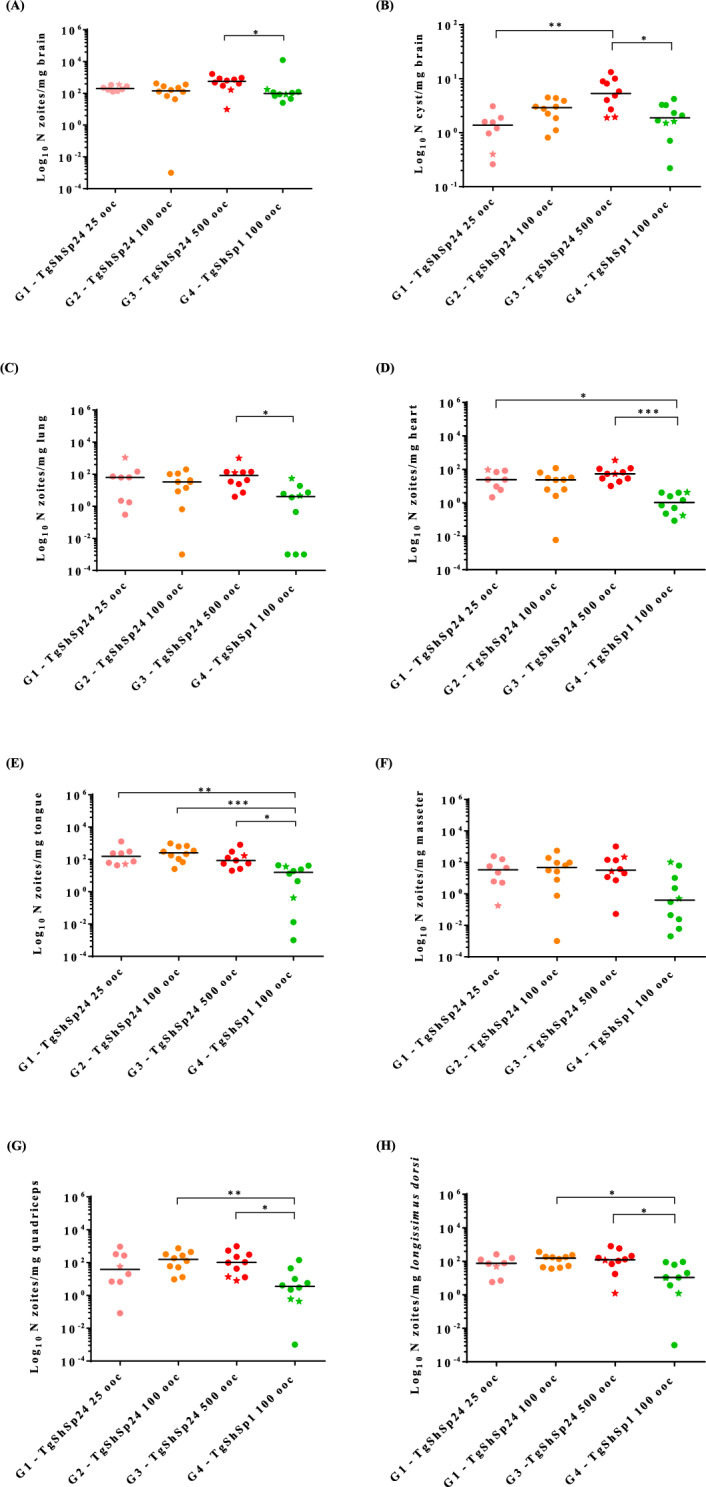
**Parasite loads in the tissues analyzed and the number of brain cysts in each mouse.** Graphs represent parasite loads recorded in the brain (**A**), lung (**C**), heart (**D**), tongue (**E**); the masseter (**F**), quadriceps (**G**), and dorsal (**H**) muscles; and cyst counts in the brain (**B**). Each point represents the number of tachyzoites or the number of cysts per milligram of tissue detected in each mouse from different infected groups (see the legend), and the transverse line represents the median. The stars indicate the mice that died early in infection: one on day 14 (infected with 25 TgShSp24 oocysts), one on day 13 (infected with 500 TgShSp24 oocysts), and one on day 18 (infected with 100 TgShSp1 oocysts). Parentheses and asterisks indicate significant differences between groups (*p < 0.05; ** p < 0.01; *** p < 0.001; Kruskal‒Wallis test followed by Dunn’s multiple comparison test). Note parasite loads and cyst counts of 0 are represented in the graphs as 0.001 and 0.1, respectively, according to the logarithmic scale of the graph. The control uninfected group with a load of 0 has not been included in the graphs.

The distribution and parasite loads determined by PCR and brain cyst counts per group are shown in Figure 2. In all infected groups, the parasite was detected in all analyzed organs and tissues, with similar detection frequencies and median parasite loads regardless of the oocyst dose (Figure 2A). Nevertheless, the brain cyst counts revealed a dose-dependent trend, with higher cyst burdens observed at increasing infection doses (Figure 2B). Spearman correlation analysis between the parasite burden determined by PCR and the number of brain cysts revealed a weak but statistically significant correlation [Spearman coefficient (ρ) = 0.54; *P* = 0.0003]. The two mice that succumbed to infection presented the highest parasite loads in the lungs and heart, intermediate loads in some skeletal muscles, and the lowest parasite burden and cyst counts in the brain among all the animals in their groups (Figure 2). These findings suggest that death occurs during the acute phase of infection. Nevertheless, the limited number of affected mice across all doses provides evidence of a predominant chronic infection following TgShSp24 exposure.

IgG serological analysis confirmed *T. gondii* infection, with no significant differences among the infected groups. However, two mice from the G1 (infected with 25 oocysts) remained seronegative at the end of the study, indicating no exposure to *T. gondii*. These mice in G1 presented no detectable parasite DNA in any tissue, confirming the absence of infection. The mouse from the same group (G1) that succumbed to the infection on day 14 post-infection also did not develop a detectable IgG response, likely due to early death. Analyses of specific IgG1 and IgG2a levels against *T. gondii* also revealed no significant differences in the IgG1/IgG2 ratios between oocyst doses.

### TgShSp24 (Type III) and TgShSp1 (Type II) isolates demonstrated differences in parasite loads and tissue tropism during chronic infection

A comparison between Type II and Type III isolates was carried out using a dose of 100 oocysts under identical experimental conditions, because the lower dose (i.e., 25 oocysts) could result in infection failure. Morbidity and clinical signs caused by the TgShSp24 isolate (G2-Type III) were more severe during the acute phase of infection (< 21 days post-infection) than those caused by the TgShSp1 isolate (G4-Type II). However, the severity of clinical signs was similar between the two groups during the chronic phase of infection (> 28 days post-infection) (Figure 1A). With respect to mortality, two mice from Group G4 were euthanized on days 18 and 25 post-infection because of the presentation of neurological signs (Figure 1C). Both mice exhibited the highest parasite loads in the heart and lungs (Figures 2C and D).

Different dissemination patterns and tissue tropisms based on parasite burdens were observed between TgShSp24 and TgShSp1 infections. The highest parasite loads were detected in mice infected with TgShSp24 (G2-Type III) compared with those infected with TgShSp1 (G4-Type II), particularly in the tongue, quadriceps and loin muscles (*p* < 0.05, Kruskal‒Wallis test followed by Dunn’s multiple comparisons test) (Figure 2). Additionally, TgShSp24-infected mice presented increased median parasite loads in the remaining target organs and tissues, suggesting better infection control in mice infected with TgShSp1 oocysts. With respect to the parasite distribution in tissues between both isolates, in the group infected with TgShSp1 (G4-Type II), the highest parasite loads were found in the brain, which were significantly greater than those in the heart, lung, masseter and quadriceps muscles (*p* < 0.05, Kruskal‒Wallis test followed by Dunn’s multiple comparisons test), followed by the tongue and dorsal muscles. In contrast, in the group infected with TgShSp24 (G2-Type III), the tongue had the highest loads, which were significantly greater than those detected in the heart and lung (*p* < 0.05, Kruskal‒Wallis test followed by Dunn’s multiple comparisons test).

The IgG responses and IgG1/IgG2a ratios of the mice in G4 were similar to those of the mice in G2 (data not shown).

## Discussion

In this study, a murine infection model based on the oral inoculation of oocysts from a Type III TgShSp24 isolate was characterized. Oocysts produced from a field isolate with minimal cell culture passages were used to avoid the consequences of laboratory adaptation on phenotypes, including virulence [8]. The advantages of this model are as follows: (i) a more natural course of infection using oocysts; (ii) the use of recently obtained field isolates, which avoids laboratory adaptation seen in strains maintained in culture; (iii) the use of a Type III isolate, as studies involving this type of infection remain limited; and (iv) the establishment of a chronic infection model that enables the study of drug and vaccine efficacy.

This model demonstrates a chronic infection profile similar to that of the Type II (TgShSp1) isolate, with no severe mortality outcomes, as expected from Type III isolates [14]. Based on the results obtained regarding sudden mortality during the acute phase, the implementation of more thorough weight monitoring of mice in future studies is recommended, particularly during the second and third weeks post-infection. This approach would allow for the detection of rapid weight loss, facilitate the identification of humane euthanasia endpoints, and minimize, as much as possible, the suffering of animals due to infection-related consequences. The results revealed a clear dose-dependent progression of clinical signs, where higher doses of TgShSp24 led to more pronounced clinical effects and increased brain cyst counts, consistent with previous findings [15]. Analyses of specific IgG1 and IgG2a levels against *T. gondii* also revealed no significant differences in IgG1/IgG2 ratios across oocyst doses, indicating no association with a Th2 or predominantly humoral response (IgG1 > IgG2a) or a Th1 or cellular response (IgG2a < IgG1). Lower doses (i.e., 25 oocysts) could result in infection failure, as found in this study and previously described for TgShSp1 [12].

Importantly, studies on infection with Type III isolates using oral inoculation of oocyst are very rare and focus mainly on the VEG isolate [5, 10]. The results obtained from the standardization of the murine model with the Type III isolate TgShSp24 revealed discrepancies compared with VEG-oocyst infection in the same Swiss Webster mouse strain [5]. For example, infection of mice with 100–1000 VEG oocysts resulted in 100% mortality [5]. In contrast, a comparable dose of 500 TgShSp24 oocysts in our study resulted in persistent infection with brain cyst formation and widespread tissue dissemination, and high parasite loads in specific muscles, but most of the mice survived the infection. *T. gondii* clonal lineages have traditionally been classified by virulence: Type I as highly virulent (100% lethality, LD_100_ = 1), Type II as intermediate (99–30%, LD_50_ ≥ 1000) and Type III as nonvirulent (< 30%, LD_50_ > 10^5^) [14]. This study aligns with the classification regarding mortality, but morbidity was greater than that associated with the Type II isolate TgShSp1. Other studies conducted with Type I or atypical isolates have shown high virulence with lethal doses as low as 1 oocyst, as traditionally reported in the literature [16, 17]. Moreover, when comparing Type II and Type III isolates, differences in morbidity and tissue cyst distribution among organs in infected animals reflect distinct pathogenic strategies, likely influenced by specific genotype‒host interactions. The differential distribution of parasite loads observed in this study aligns with previous research, which indicates that Type II strains tend to exhibit specificity for the brain and more restricted systemic dissemination than do Type III strains [13, 18, 19]. In addition, Type III strains tend to be more virulent and produce greater burdens in target organs such as the brain and muscles [4, 13, 18].

Recent studies using oocysts from the same *T. gondii* isolate have been carried out to evaluate infection dynamics and standardize models in target species such as piglets. This type of infection provides a more natural route that closely mimics real-world transmission [13]. A TgShSp24 Type III murine infection model correlates with a toxoplasmosis model in piglets. Similar results were observed in the piglet model, particularly the more pronounced clinical manifestations caused by the TgShSp24 isolate, including higher and longer-lasting fevers during the acute phase, followed by partial recovery in the chronic phase [13]. These findings align with observations in the mice in this study, where higher clinical scores were recorded during the acute phase with TgShSp24. Moreover, the detection of the parasite by PCR in both models revealed a similar distribution across target tissues, as well as higher parasite loads in tissues from animals infected with the TgShSp24 isolate [13].

In conclusion, the phenotypic variability of these field isolates may influence infection outcomes, pathogenic mechanisms, and therapeutic approaches, underscoring the relevance of this new model. The murine infection model developed in this study more closely reflects natural routes of transmission, as it involves infection with a recently obtained isolate and the oocyst stage. The observed similarities also validate the extrapolation of the results from mice to piglets, positioning the mouse as an efficient and cost-effective model for the preliminary screening of drugs or vaccines against *T. gondii*. This approach optimizes resources and reduces reliance on large animal studies, representing a significant innovation in the preclinical development of control strategies for this zoonosis.

## Data Availability

All data generated or analyzed during this study are included in this published article. Further inquiries can be directed to the corresponding authors.
